# Electrospun Hybrid Perfluorosulfonic Acid/Sulfonated Silica Composite Membranes

**DOI:** 10.3390/membranes10100250

**Published:** 2020-09-23

**Authors:** Leslie Dos Santos, Devon Powers, Ryszard Wycisk, Peter N. Pintauro

**Affiliations:** Department of Chemical and Biomolecular Engineering, Vanderbilt University, Nashville, TN 37235, USA; dossantos.leslie@gmail.com (L.D.S.); devon.j.powers@vanderbilt.edu (D.P.); ryszard.wycisk@vanderbilt.edu (R.W.)

**Keywords:** electrospinning, nanofibers, sol-gel silica, fuel cell membranes

## Abstract

Electrospinning was employed to fabricate composite membranes containing perfluorosulfonic acid (PFSA) ionomer, poly(vinylidene fluoride) (PVDF) reinforcement and a sulfonated silica network, where the latter was incorporated either in the PFSA matrix or in the PVDF fibers. The best membrane, in terms of proton conductivity, was made by incorporating the sulfonated silica network in PFSA fibers (Type-A) while the lowest conductivity membrane was obtained when sulfonated silica was incorporated into the reinforcing PVDF fibers (Type-B). A Type-A membrane containing 65 wt.% PFSA with an embedded sulfonated silica network (at 15 wt.%) and with 20 wt.% PVDF reinforcing fibers proved superior to the pristine PFSA membrane in terms of both the proton conductivity in the 30–90% RH at 80 °C (a 25–35% increase) and lateral swelling (a 68% reduction). In addition, it was demonstrated that a Type-A membrane was superior to that of a neat 660 EW perfluoroimide acid (PFIA, from 3M Co.) films with respect to swelling and mechanical strength, while having a similar proton conductivity vs. relative humidity profile. This study demonstrates that an electrospun nanofiber composite membrane with a sulfonated silica network added to moderately low EW PFSA fibers is a viable alternative to an ultra-low EW fluorinated ionomer PEM, in terms of properties relevant to fuel cell applications.

## 1. Introduction

One of the key challenges for proton-exchange membrane (PEM) fuel cell development is the fabrication of a membrane with high proton conductivity for a wide range of relative humidity (RH), while having good mechanical strength, and low in-plane selling [1,2,3,4]. The benchmark membrane materials for H_2_/air fuel cells are perfluorosulfonic acid (PFSA) ionomers, e.g., Nafion^®^ from Chemours and Aquivion^®^ from Solvay [5]. Membranes fabricated from these ionomers have been shown to exhibit high proton conductivity at 100% relative humidity, as well as good chemical and mechanical stability during fuel cell operation. These outstanding properties arise from the superacidity of sulfonic acid fixed-charge groups (in the terminal positions on ether-linked side chains), the semi-crystalline nature of moderate/high equivalent weight (EW) PFSAs, and the nano-phase segregated morphology under hydrated conditions, where ionic clusters aggregate to form water channels which are separated from the hydrophobic polytetrafluoroethylene backbone domains [6,7]. Unfortunately, the proton conductivity of these membranes decreases dramatically at low RH due to disruption of the ionic network and reversal of sulfonic group dissociation, which reduce the proton concentration within the membrane.

One common strategy to increase membrane conductivity at low humidity is to utilize a high ion-exchange capacity (IEC, with units of mmol/g) ionomer. For example, 3M Company has developed a broad range of short side chain PFSA ionomers with equivalent weights as low as 580 g/mol [8,9,10] (the equivalent weight is defined as the polymer weight per mole of ion-exchange groups and is related to IEC by the simple equation EW = 1000/IEC). Unfortunately, around 700 EW, PFSA polymer crystallinity disappears and the resultant membrane swells excessively when hydrated, and exhibits a loss in mechanical strength. At an even lower equivalent weight (650 EW or less), the ionomer becomes soluble in hot and then in cold water. To counteract the water solubility problem 3M Co. has developed a new series of low EW, water insoluble perfluoroimide acid (PFIA) ionomers where there are two proton-exchange groups on each polymer sidechain, a sulfonic acid and a sulfonimide moiety [10]. While these ionomers, e.g., EW 580 PFIA, exhibit good conductivity in the RH range of 50–100%, they have been shown to degrade in the presence of peroxides and hydroxyl radicals [10].

An alternative approach to increasing an ionomer’s IEC by covalent attachment of additional charged groups is the addition of inorganic, proton-conducting materials such as sulfonated silica [11,12,13,14,15,16,17], zirconium phosphate [18,19,20,21,22,23] or a heteropolyacid [24,25,26]. Such hybridized (composite) membranes exhibit improved water retention and conductivity, and frequently, improved thermal and chemical stability. For example, Choi et al. [27,28] developed a nanofiber-based polymer/particle composite membrane with very high conductivity, even at low RH. The membrane was fabricated from electrospun nanofibers composed of sulfonated poly(arylene ether sulfone) or 825 EW PFSA, where sulfonated polyhedral oligomeric silsesquioxane (sPOSS, with an IEC of 4.8 mmol/g) was added to the ionomer fibers as a proton conductivity enhancer. The ionomer/sPOSS fibers were embedded in a matrix of Norland Optical Adhesive (an uncharged photo-crosslinkable polyurethane) to create a pore-free (dense) film. In such a membrane, proton-conducting nanofibers are physically separated from the uncharged polymer, where the latter material controls both the mechanical strength of the membrane and the membrane’s dimensional stability when equilibrated in water. For a PFSA/sPOSS nanofiber composite membrane, the proton conductivity at 120 °C and 50% RH was 2.5 times higher than that of Nafion (0.107 vs. 0.039 S/cm). Unfortunately, membrane conductivity decreased over time as the sPOSS particles slowly leached out of the membrane. More recently, Laberty-Robert et al. [29,30,31] described the fabrication of organic/inorganic membranes composed of a functionalized silica network with sulfonic acid groups, embedded in a hydrophobic polymer—poly(vinylidene fluoride-co-hexafluoropropylene). Membranes were prepared by combining in-situ sol-gel chemistry and electrospinning. This novel approach allowed for intimate mixing of hydrophobic and hydrophilic membrane components at the nanoscale. The sol-gel precursor (silane mixture) was pre-hydrolyzed before electrospinning, and the resultant mat was dried at elevated temperature to assure complete condensation and insolubility of the siloxane network. Such a membrane exhibited a conductivity of 15 mS/cm at 120 °C under 50% relative humidity and a modulus greater than that of Nafion at temperatures > 80 °C.

**Figure 1:**
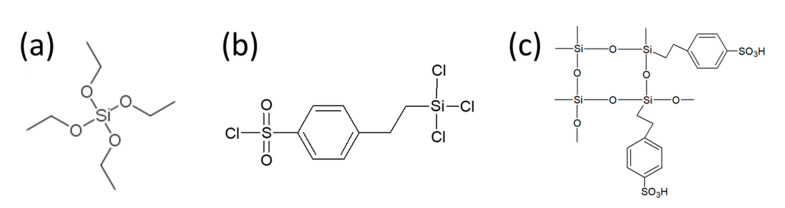
Silica precursors used in this study: (**a**) TEOS (tetraethyl orthosilicate), and (**b**) CSPTC (2-(4-chlorosulfonylphenyl)ethyltrichlorosilane). The sulfonated silica network after sol-gel hydrolysis and polycondensation is shown in (**c**).

In the present work, the above strategy has been expanded by combining dual fiber electrospinning and in-situ sol-gel chemistry for the fabrication of hybrid nanofiber composite proton-conducting membranes. The membranes were made from co-electrospun 3M 825 EW perfluorosulfonic acid (PFSA) and poly(vinylidene fluoride) (PVDF) mixed-fiber mats, which also contained a highly conductive inorganic sulfonated silica network generated by sol-gel co-condensation of tetraethyl orthosilicate (TEOS) and (4-chlorosulfonylphenyl)ethyltrichlorosilane (CSPTC), in either the PFSA or the PVDF fibers (Figure 1). After electrospinning, the mats were densified by hot-pressing, which softened the ionomer and eliminated porosity, to obtain structures with an ionomer matrix and an embedded network of reinforcing PVDF nanofibers. Ballengee and Pintauro [32] showed that this type of composite structure leads to an ion conducting membrane with low in-plane swelling and good mechanical properties. In the present paper, the conductivity, water swelling, and mechanical properties of the two types of hybrid nanofiber composite membrane (with sol-gel sulfonated silica in either the PFSA or PVDF fibers) were analyzed and compared to those of a nanofiber composite membrane without sulfonated silica. The objective was to identify which structure was the most effective in improving the original PFSA membrane characteristics critical for fuel cell operation, focusing on increasing proton conductivity at low RH and reducing lateral swelling in water.

## 2. Materials and Methods

825 EW perfluorosulfonic acid (PFSA) and poly(vinylidene fluoride) Solef 6020-1001 PVDF were supplied by 3M Company (Saint Paul, MN, USA) and Solvay Specialty Polymers (Alpharetta, GA, USA), respectively. Tetraethyl orthosilicate (TEOS), poly(ethylene oxide) (PEO, with a MW of 600 kDa), *N,N*-dimethylacetamide 99.8% (DMAc), n-propanol, and acetone were purchased from Sigma Aldrich and used as received. 2-(4-chlorosulfonylphenyl)ethyltrichlorosilane (CSPTC), as a 50% methylene chloride solution, was purchased from Gelest, Inc. (Morrisville, NC, USA).

### 2.1. Nanofiber Electrospinning

Nanofiber composite membranes were fabricated by simultaneously electrospinning 825 EW PFSA and PVDF fibers from separate needle syringe spinnerets onto a common rotating drum collector. Inorganic precursors were added to the PFSA dispersion or to the PVDF solution before electrospinning. Two inorganic precursors were used to create the highly conductive functionalized silica network: 2-(4-chlorosulfonylphenyl)ethyltrichlorosilane (CSPTC) for proton conductivity and tetraethyl orthosilicate (TEOS) to optimize the crosslinking of the inorganic network. The CSPTC/TEOS mass ratio was kept constant at 6/1, which corresponded to an ion exchange capacity (IEC) of 4.4 mmol/g for the silica network. Three types of nanofiber composite membranes were fabricated by dual fiber electrospinning:

Type-A Dual Fiber Mats: 65-15/20 (wt.-wt./wt.) 825 EW PFSA-SiO_x_SO_3_H/PVDF fiber mats were prepared and eventually transformed into a membrane where the structure was a PFSA-SiO*x*SO_3_H matrix with an embedded network of uncharged PVDF reinforcing nanofibers. The dual fiber mats were prepared from two separate electrospinning solutions. 3M 825 EW PFSA and PEO (*M*w = 600 kDa) were separately added to a 2/1 weight ratio n-propanol/water mixed solvent. The PEO fully dissolved, whereas the PFSA formed a clear micellar solution. PEO was used here to increase chain entanglement which enabled nanofiber electrospinning of the PFSA dispersion [33,34,35,36]. The PEO solution was then added to the PFSA dispersion, resulting in a mixture that contained 20 wt.% polymer with a 99/1 (wt./wt.) ratio of 825 EW PFSA/PEO. TEOS was added to the dispersion with 100 µL of 0.2 M HCl to assure acidic conditions, and the solution was mixed for 2 h. Finally, CSPTC was added and the solution was mixed for 1 h at 70 °C for pre-hydrolysis of the inorganic network. The solution was allowed to cool to room temperature before electrospinning. The mass ratio of CSPTC/TEOS was 6/1 and the PFSA/SiO_x_SO_3_H was 70/30 wt./wt. before hydrolysis. A separate solution of PVDF was prepared by dissolving polymer into a 9/1 wt./wt. mixture of DMAc/Acetone to a final polymer content of 17.5 wt.%. After simultaneously electrospinning PFSA-SiO*_x_*SO_3_H and PVDF fibers, the resulting mat was dried at 70 °C overnight. The final composition of the mat was 65-15/20 (wt.-wt./wt.) 825 EW PFSA-SiO_x_SO_3_H/PVDF. The electrospinning conditions for this type of fiber mat are listed in Table 1. In a similar way, two additional Type-A mats were prepared, in which the sulfonated silica content was 9 wt.% and 26 wt.%, where the PVDF content was kept constant at 20 wt.%.

Type-B Dual Fiber Mats: Fiber mats with a composition of 65/15-20 (wt./wt.-wt.) 825 EW PFSA/SiO*_x_*SO_3_H-PVDF where prepared and transformed into membranes where the PFSA matrix contained an embedded network of reinforcing fibers composed of PVDF-SiO*_x_*SO_3_H. The ionomer electrospinning solution for these mats was prepared by mixing separately 3M 825 EW PFSA and PEO in 2/1 weight ratio n-propanol/DI water. The dissolved PEO was then added to the 825 EW PFSA dispersion for a PFSA/PEO mass ratio of 99/1, where the total polymer concentration was 20 wt.%. The PVDF electrospinning solution was prepared in a mixture of DMAC/Acetone (9/1 wt./wt.) at a polymer concentration of 8 wt.%. TEOS was added to this solution with 100 µL of 0.2 M HCl to assure acidic conditions, followed by 2 h of mixing. Finally, CSPTC was added and the solution was mixed at 70 °C for one hour. The CSPTC/TEOS mass ratio was 6/1 and the PVDF/SiO*_x_*SO_3_H is 40/60 wt./wt. before hydrolysis. The electrospinning conditions for the dual fiber mat are summarized in Table 1. The mat was dried at 70 °C overnight before further processing into a dense membrane, as will be described below.

Type-C Dual Fiber Mat: 80/20 (wt./wt.) 825 EW PFSA/PVDF dual fiber mat that will be converted into a reference membrane, where a PFSA matrix is embedded with a network of uncharged PVDF reinforcing nanofibers. The mat was made using a procedure similar to that for a Type-A dual fiber mat, but with no added silica. 825 EW PFSA and PEO were separately added to n-propanol/DI water solvent. The PEO solution was then added to the PFSA dispersion to give a PFSA/PEO ratio of 99/1 (wt./wt.), where the total polymer concentration was 20%. The PVDF solution was prepared by dissolving polymer in a mixture of DMAC/Acetone (9/1 wt./wt.) to a polymer concentration of 17.5 wt.%. PFSA and PVDF fibers were simultaneously electrospun onto a common collector and dried overnight at 70 °C. The electrospinning conditions are summarized in Table 1.

### 2.2. Transforming Dual Fiber Mats into Dense Membranes

Mats A, B, and C were converted into dense and defect-free membranes by hot-pressing and annealing. Hot-pressing was carried out at 143 °C and 7000 psi for 5 min. At this temperature, the PFSA fibers were selectively softened while the PVDF maintained its fiber structure. Annealing was done in a vacuum oven at 200 °C for 30 min. The resultant membranes had a PFSA matrix with embedded PVDF reinforcing fibers (all nanofiber composite membranes had this structure). Such a membrane morphology (an uncharged polymer fiber network embedded in an ionomer matrix) was shown to exhibit low in-plane swelling [32], which was identified as critical for long-term membrane durability in a fuel cell [1]. Sol-gel-containing membranes were immersed in 0.1 M NaOH at room temperature for 1 h to ensure complete hydrolysis/condensation of the inorganic network components. Membranes were pretreated by soaking in 1.0 M H_2_SO_4_ at 80 °C for 1 h and then in DI water at 80 °C for 1 h to insure that all sulfonic acid groups were in the proton form. Membranes were equilibrated/stored in DI water at room temperature before testing. Membranes were typically 15–40 μm in dry thickness.

### 2.3. Fabrication of a Solution-Cast Blended Membrane (Membrane Type-D)

A solution cast blend membrane (hereafter identified as Membrane Type-D) with the same overall composition as Membranes A and B: 65/15/20 (wt./wt./wt.) 825 EW PFSA/SiO*_x_*SO_3_H/PVDF, was also fabricated for comparison to the electrospun films. PVDF and PFSA were separately pre-dissolved in DMAC, and then the two solutions were mixed together. The polymer concentration was 10 wt.%. TEOS was added with 100 µL of 0.2 M HCl and the dispersion was mixed at room temperature for 2 h. CSPTC was added with an additional 1 h of mixing at 70 °C. Finally, the solution was cooled to room temperature, cast onto a clean glass plate, and allowed to dry at 70 °C overnight. The film was annealed for 30 min at 200 °C in a vacuum oven and the membrane was pretreated by soaking for 1 h in 1.0 M H_2_SO_4_ at 80 °C, washing for 1 h with DI water at 80 °C, and then equilibrating in room temperature DI water.

### 2.4. Characterization of Fiber Mats and Membranes

#### 2.4.1. Scanning Electron Microscopy

Surfaces of electrospun mats and membranes were imaged with a Hitachi S-4200 scanning electron microscope (Tokyo, Japan). Samples were sputter-coated with a gold layer (≈ 5 nm) to prevent charging of the specimen and to reduce thermal damage while improving secondary electron emission.

#### 2.4.2. Ion-Exchange Capacity (IEC)

The ion-exchange capacity (IEC) of a membrane was determined using an acid/base titration technique. A membrane sample of known dry weight was equilibrated in 1.0 M H_2_SO_4_ and then thoroughly washed in DI H_2_O over a period of several hours. The membrane was then immersed in a 2.0 M NaCl solution for at least 24 h to exchange H^+^ fixed-charge site counterions with Na^+^. After removing the membrane, the soak solution was titrated with 0.01N NaOH to a neutral pH. The IEC of the membrane was calculated with the following equation:

(1) IEC=1000×(V×Nmd)

where *V* (mL) is the volume of titrant required to bring the soak solution to a neutral pH, *N* (mol/L) is the normality of the titrant, and *m_d_* (g) is the dry mass of the membrane.

#### 2.4.3. Proton Conductivity

In-plane proton conductivity was determined using an *ac*-impedance method and a BekkTech four-electrode test cell [37]. Although the through-plane conductivity is most relevant to real-world membrane applications, the measurement is difficult to perform and prone to significant experimental errors, associated with estimating surface impedance at the membrane/electrode interfaces. For this reason, the vast majority of proton conductivity measurements reported in the literature, including those in the present study have been performed in the in-plane direction. The BekkTech cell was either immersed in room temperature (20 °C) water or placed in an ESPEC Corp. temperature/humidity controlled environmental chamber (Model: SH-241) for testing at 80 °C and a relative humidity between 20% and 90%. In-plane conductivity was calculated using the following equation:

(2) $$\sigma =\frac{L}{Rw\delta }$$

where *σ* (S/cm) is proton conductivity, *R* (Ω) is the resistance (real axis intercept on Nyquist plot), *L* (cm) is the distance between the inner electrodes, *w* (cm) is the width of a membrane sample (typically 0.5 cm), and *δ* (cm) is the thickness of the sample (typically between 0.0015 and 0.0040 cm). For conductivity calculations of water-equilibrated samples, the swollen membrane dimensions were used, whereas air-dried dimensions were used for the conductivity of vapor-equilibrated samples. As a check on reproducibility, conductivity measurements were repeated on duplicate membrane samples. Typical experimental errors were no more than 8%.

#### 2.4.4. Gravimetric Water Uptake and In-Plane Swelling

Gravimetric water uptake and in-plane swelling were determined after membrane equilibration in room temperature DI water. Excess water was gently removed from the membrane surface with filter paper after equilibration and then the wet mass and the length were measured. Sample was then dried for 12 h in vacuum at 70 °C and for 1h under vacuum at 100 °C, and the weight and length measurements were retaken. Gravimetric water uptake and in-plane swelling were calculated using the following equation:

(3) $$Water\;Uptake\;\left(g/g\right)\;or\;In-plane\;Swelling\;\left(\%\right)=\;\frac{x_{wet}-x_{dry}}{x_{dry}}\times 100$$

where *x* is the membrane mass (for water uptake) or length (for in-plane swelling) in the wet or dry states. The variation in in-plane and gravimetric water uptake from duplicate measurements was about ±5%.

#### 2.4.5. Water Vapor Sorption

The gravimetric uptake of water vapor was measured using a dynamic water-vapor sorption analyzer (model Q5000SA, TA Instruments, New Castle, NY, USA). Each membrane sample was pre-dried at 80 °C and 0% RH for ca. 200 min, until the sample weight stabilized, i.e., until the measured weight change over a 5 min time interval was less than 0.001%. The humidity was then slowly increased from 10% to 90% RH at a step-size of 10%. Sample equilibration (weight stabilization) at a given humidity typically required no more than 100 min.

#### 2.4.6. Membrane Mechanical Properties

The stress at break of membrane samples was measured with a TA Instruments Q800 dynamic mechanical analyzer (DMA, TA Instruments, New Castle, NY, USA). Stress-strain curves were obtained for membranes equilibrated in air at 22 °C and ≈ 20% RH after the samples were pre-dried in vacuum at 60 °C for 12 h. The DMA was operated in tension using the controlled strain mode, where the sample was strained at 10%/min until failure.

## 3. Results

**Figure 2:**
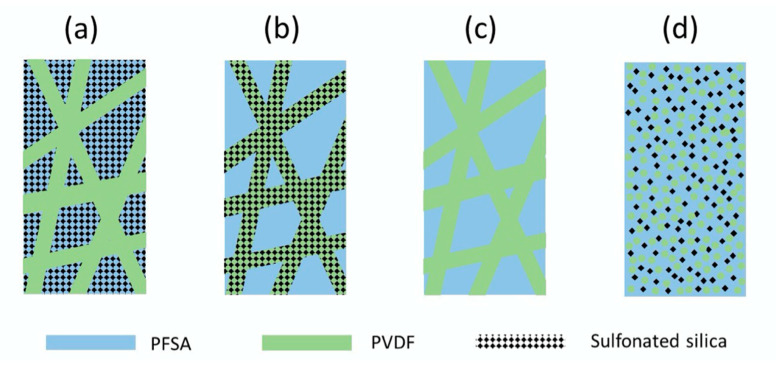
Schematic representations of the membranes fabricated in this study. (**a**) Membrane Type-A, (**b**) Membrane-Type-B, (**c**) Membrane Type-C, and (**d**) Membrane Type-D.

Four types of composite membranes were fabricated and characterized: (1) Membrane Type-A had a PFSA-SiO*_x_*SO_3_H matrix with an embedded network of uncharged PVDF reinforcing nanofibers, (2) Membrane Type-B was made with a PFSA matrix having an embedded network of reinforcing fibers composed of PVDF-SiO*_x_*SO_3_H, (3) Membrane Type-C (a reference film) consisted of a PFSA matrix with an embedded network of uncharged PVDF reinforcing nanofibers (no SiO*_x_* added), and (4) Membrane Type-D, which was a solution cast blended membrane with PFSA, PVDF and SiO*_x_*SO_3_H. Membrane Types A, B, and D had the same overall composition: 65/15/20 wt./wt./wt. 825 EW PFSA/SiO*_x_*SO_3_H/PVDF. For clarity, the morphologies of the four membrane types are shown schematically in Figure 2.

### 3.1. Preliminary Studies with Type-A Membranes

An initial set of Type-A membrane fabrication experiments were carried out to determine how the replacement of a certain fraction of PFSA with sulfonated silica affects the membrane fibrous structure, IEC, proton conductivity and water uptake. The compositions of four different Type-A fiber mats and final membranes are listed in Table 2. The sulfonated silica content was determined by assuming complete condensation of the TEOS and CSPTC network, which was the theoretical maximum for a given membrane’s silica content. As shown in the table, there is approximately a 50% reduction in the inorganic component content due to a loss of the ethyl groups of TEOS upon complete condensation (for example, if 30 wt.% inorganic sol-gel material was added to the PFSA electrospinning solution, there was a weight increase of ~15 wt.% in the final membrane due to the presence of sulfonated silica material). It was also found that without the final NaOH soaking step, some unreacted silane leached out from the membranes during pretreatment in boiling acid and water, due to incomplete condensation of the TEOS/CSTPC network.

Experimental and theoretical ion-exchange capacities (IECs) of these membranes are listed in Table 3. As expected, the addition of the inorganic sulfonated silica network increased the composite membrane IEC, compared to the Type-C reference membrane. The close match of the experimental and theoretical IEC values for nanofiber composite membranes with 9 wt.% and 15 wt.% SiO*_x_*SO_3_H indicated excellent hydrolytic stability of the sulfonated silica network during the acid/water boiling pretreatment steps, even though its IEC is very high (an estimated IEC of 4.4 mmol/g). The drop in membrane IEC, when the sulfonated silica content was increased from 15 wt.% to 26 wt.% (both before and after the NaOH soak), is attributed to precipitation/agglomeration of the sol-gel precursors in the PFSA fibers, with the formation of sulfonated silica nanoparticles rather than a distributed silica network, where the former leached out of the membrane after immersion in water. Such leaching of sulfonated silica nanoparticles was observed in a previous electrospun composite membrane study [28].

**Figure 3:**
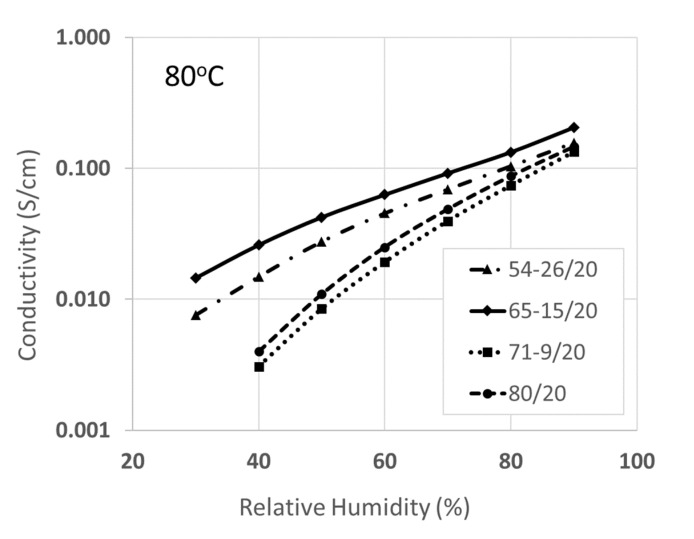
In-plane proton conductivity at 80 °C versus relative humidity for Type-A hybrid membranes, where a SiOxSO_3_H network was incorporated into the PFSA matrix. The PFSA-SiOxSO_3_H/PVDF weight percent composition of the membranes is given in the insert (the 80/20 film has no SiOxSO_3_H).

The dependence of proton conductivity on RH at 80 °C for the four membranes in Table 3 is shown in Figure 3. The most conductive membrane over the entire humidity range contained 15 wt.% SiO*_x_*SO_3_H (as expected, based its high IEC in Table 3), with a more pronounced improvement over the 80/20 Type-C nanofiber composite film (no added sulfonated silica) as the relative humidity decreased. At 90% RH, the two Type-A membranes containing 9 and 26 wt.% SiO*_x_*SO_3_H and the Type-C membrane had a similar conductivity, about 0.15 S/cm. Surprisingly, the membrane containing 9 wt.% sulfonated silica had a conductivity lower than the Type-C membrane (with no sulfonated silica) over the entire humidity range (40–90% RH), even though its IEC was about 40% greater than that of the Type-C membrane. The lower-than-expected conductivity was associated with the combined effects of the lower acidity of the aryl sulfonic acid moieties in the sulfonated silica network, as compared to that of the super-acidic perfluorosulfonic acid groups in PFSA, and insufficient percolation of the sulfonated silica network through the composite film. For the membrane with 26 wt.% sulfonated silica, its lower than expected conductivity was consistent with its low IEC, where there was partial leaching of sulfonated silica out of the membrane after the samples was pre-treated in acid and water. Although a fraction of the sulfonated silica was lost, the remaining amount was still sufficient to provide enhanced conductivity at low humidity, and thus the membrane had a higher conductivity than the hydrolytically stable film with 9 wt.% sulfonated silica, even though its IEC was smaller.

### 3.2. Type-A Membrane vs. Type-B Membrane—Location of Sulfonated Silica

Once the characteristics of Type-A membranes were examined, the question was asked: Would it be even more beneficial to embed sulfonated silica in the reinforcing PVDF fibers and not in the PFSA matrix? The most conductive, Type-A membrane, had 15 wt.% sulfonated silica and so a Type-B membrane was also prepared with 15 wt.% sulfonated silica, but the silica network was located within the PVDF fibers. To complete the analysis, a non-fibrous Type-D membrane was fabricated by solution casting a membrane, where the final membrane morphology was a blend of PFSA and PVDF with 15 wt.% sulfonated silica intermixed within the blend.

**Figure 4:**
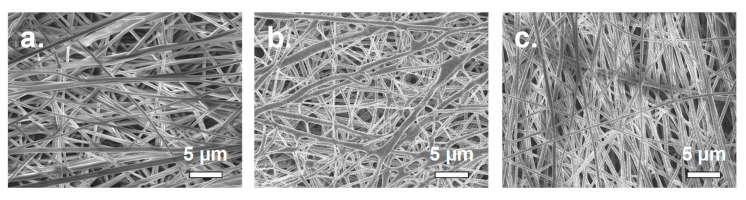
Scanning electron microscopy images of a mixed fiber mat where: (**a**) the inorganic sulfonated silica network was incorporated into the PFSA fibers (Mat Type-A), (**b**) the inorganic network was incorporated into PVDF fibers (Mat Type-B), and (**c**) no inorganic network was added to either fiber type (Mat Type-C).

Figure 4a,b show top down SEM images of electrospun Type-A and Type-B. Well-formed fibers were obtained when the inorganic sulfonated silica network was incorporated into the PFSA fibers (Type-A mat in Figure 4a), whereas extensive inter-fiber welding was observed when the silica was incorporated into PVDF fibers (Type-B mat in Figure 4b), for reasons not well understood at this time. SEM image of the electrospun Type-C mat, with PFSA/PVDF fibers and no sulfonated silica is shown in Figure 4c. The PFSA and PVDF fibers are indistinguishable, with an average diameter of 398 ± 48 nm, which is smaller than that for both, Type-A fibers (473 ± 50 nm) and Type-B fibers (558 ± 92 nm). The increase in average fiber diameter was associated with the fibers containing sulfonated silica (the SiO*_x_*SO_3_H network).

**Figure 5:**
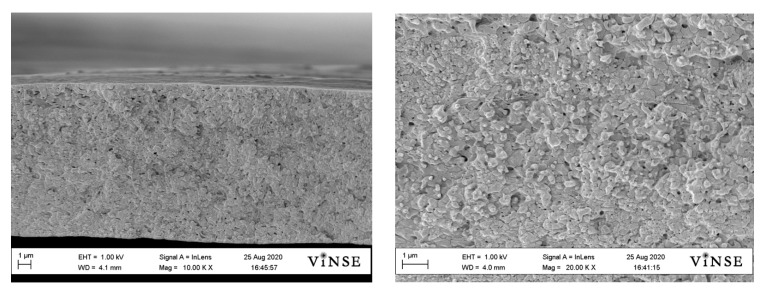
Low (**left**) and high (**right**) magnification SEM images of freeze-fractured cross sections of a fully processed membrane (Membrane Type-A).

Representative SEMs of freeze-fractured membrane cross-sections after processing the dual fiber mats into dense membranes is shown in Figure 5 for a Type-A membrane. The void volume between fibers, as shown in Figure 4a was eliminated, with a uniform distribution of reinforcing PVDF fibers across the entire film. Such a dense morphology was typical for all of the membranes evaluated in this study. What appear to be small pores in the membrane are actually artifacts of the freeze-fracturing procedure, where PVDF reinforcing fibers are pulled away from the freeze-fractured surface.

Compositions and selected properties are listed in Table 4 for the three PFSA/sulfonated silica membranes (Types A, B, and D), the silica-free electrospun composite film (Membrane Type-C), and a neat solution cast film of 825 EW PFSA (Membrane Type-E). Adding the SiO*_x_*SO_3_H network to either the PFSA fibers or PVDF fibers lead to the same increase in the membrane ion-exchange capacity vs. Membrane Type-C. Thus, even though the sulfonated silica was embedded in the highly hydrophobic PVDF fibers there was still a sufficient number of hydrated, percolating contacts so that access of water and ions to the SiO_x_SO_3_H network was not inhibited. Moreover, the experimentally determined IECs of the membranes given in Table 4 match the anticipated IEC of 1.45 mmol/g, calculated based on the mats’ composition.

Several additional conclusions can be drawn based on the Table 4 data. First, the decrease in IEC and proton conductivity for the 80/20 PFSA/PVDF nanofiber composite membrane, as compared to the neat 825 EW film, is due to the addition of PVDF (a dilution effect where the proton conductivity decreases with PFSA according to a linear mass-fraction mixing rule). Second, no change in proton conductivity (in liquid water) of a fiber composite film was observed when inorganic sulfonated silica was added to the PFSA matrix (0.089 S/cm for Membrane Type-A vs. 0.087 S/cm for Membrane Type-B). This surprising result suggests that the sulfonated silica network is not contributing to proton conduction when the membranes are equilibrated in liquid water (as will be shown below, the proton conductivity is very different for films equilibrated in water vapor). Third, there was no increase in gravimetric or in-plane water swelling between Type-A and Type-C membranes, which is in direct contrast to the results of Choi et al. [27] who found that the addition of sPOSS (sulfonated silica) particles to 825 EW PFSA resulted in a higher membrane water content. In this regard, the results in Table 4 suggest that the SiO*_x_*SO_3_H network provides added mechanical strength/stability to the membrane, thus limiting water swelling, which does not occur when discrete sulfonated silica particles (sPOSS) are incorporated into the film. The control of membrane swelling is further exemplified by the fact that the gravimetric swelling of Membrane Type-A was the same as a neat PFSA film, even though its ion-exchange capacity (hydrophilicity) was higher than that for an 825 EW ionomer. Fourth, to our surprise, there was a dramatic decrease in proton conductivity as compared to that of the reference film when the SiO*_x_*SO_3_H network was located in the PVDF fibers (Membrane Type-B) or distributed (presumably uniformly) throughout a solution-cast blended film (Membrane Type-D). Although the sulfonic acid groups in both films were accessible to water and were fully dissociable (the membranes had a similar IEC), such groups did not contribute to proton conductivity and actually caused the membrane conductivity to drop below that for a silica-free 80/20 PFSA/PVDF membrane. For Membrane Type-B, this could be associated with trapping of some water at sulfonated silica sites within the PVDF fibers, thus reducing the amount of water in the PFSA matrix (where there was insufficient water present for complete dissolution of all sulfonic acid sites in PFSA and/or an insufficient number of water-filled channel for facile proton migration). For the solution cast membrane with a sulfonated silica network (Membrane Type-D), water was also playing a dominant role on conductivity. The swelling of this film was very low (as has been observed previously in solution-cast blended films [38]), leading to poor hydration of the SiO_x_SO_3_H domains and/or an insufficient number of water channels.

**Figure 6:**
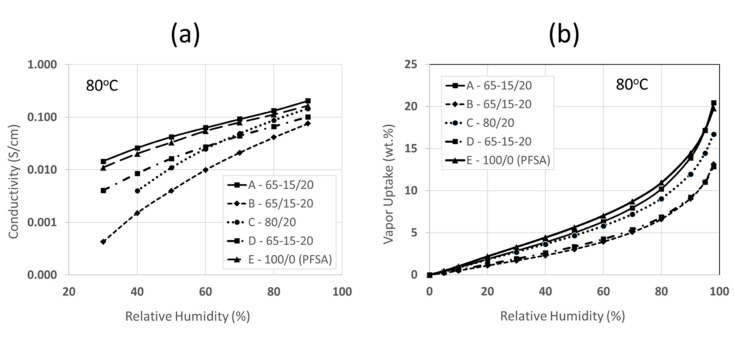
(**a**) In-plane proton conductivity at 80 °C versus relative humidity for the five membranes listed in Table 4 (Type-A through Type-E); (**b**) Gravimetric equilibrium water vapor sorption isotherms at 80 °C for the same five membranes.

The dependence of proton conductivity (in-plane) on relative humidity (RH) at 80 °C is shown in Figure 6a for the five membranes in Table 4. Membrane Type-A has the highest conductivity for the entire 30–90% RH range. At 30% RH, its conductivity (0.015 S/cm) is 37.5 times higher than that of a nanofiber composite membrane with sulfonated silica in PVDF fibers (Membrane Type-B, with a conductivity of 0.0004 S/cm), and 2.7 times greater at 90% RH (0.206 vs. 0.076 S/cm). Membrane Type-E (a solution cast 825 EW PFSA film) showed reasonably good conductivity at 40% RH (about 75% that of Membrane Type-A) and at 90% RH (80% that of Membrane Type-A). The conductivities of Membranes Type-C and Type-D were positioned somewhere midway between those of Type-A and Type-B. Membrane Type-D was more conductive than membrane Type-C at the lower RH limit while Membrane Type-C performed better at 90% RH (as it did in liquid water as per Table 4), with a crossover point at ~60% RH. While the reduction in conductivity of Membrane Type-C with respect to that of Type-E was expected (20 wt.% of PFSA was replaced with nonconductive PVDF fibers in the former), it was surprising to observe the relatively low conductivity in Membrane Type-D (solution cast blend), but the poor RH results are consistent with the low conductivity after equilibration of this film in liquid water, as shown in Table 4.

The conductivity results appear to be consistent with water vapor sorption data (shown in Figure 6b), where Membrane Type-A and a neat 825 EW PFSA film exhibit the greatest water uptake at 80 °C, while Membrane Type-D (the solution cast blend of PFSA/SiO*_x_*SO_3_H/PVDF) sorbed the least. The high water content but low conductivity of the neat PFSA film, as compared to the nanofiber composite membrane with sulfonated silica (Membrane Type-A) exemplifies the importance of controlling/lowering membrane water swelling when increasing the membrane IEC, i.e., the IEC is based on the dry polymer weight and does not take into account the separation distance between fixed charge sites in a water-swollen membrane.

**Figure 7:**
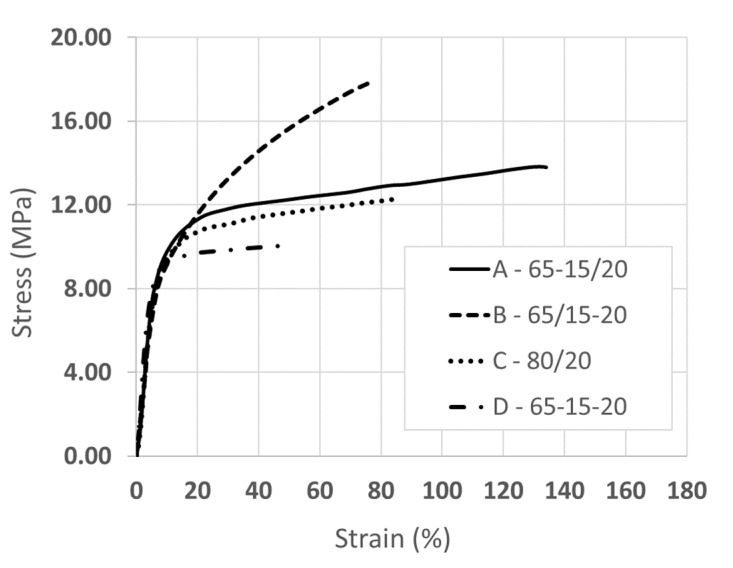
Stress-strain curves for Membrane Types A-D at 22 °C and 20% RH.

Stress-strain curves for nanofiber composite membranes (Type-A, Type-B and Type-C) and the solution-cast blend membrane (Type-D) are shown in Figure 7, and the extracted values of the ultimate stress and strain, and modulus are compared in Table 5.

The incorporation of a sol-gel sulfonated silica network (Membrane Type-A, Type-B and Type-D) resulted in an increase in the stress at break, as compared to that of a nanofiber composite membrane without the silica network. The composite membrane with sulfonated silica in PVDF fibers exhibited the greatest stress at break (18 MPa). Type-A membrane has the highest strain at break (134%) and modulus (152 MPa). Additionally, within 5% to 10% elongation, its tensile stress was the greatest among the four membrane types. The increase in mechanical strength of the two hybrid sol-gel nanofiber membranes is associated with the stiffness of the inorganic network formed within the PFSA or PVDF fibers during electrospinning. The poor strength and low elongation at break of the solution cast film with added sol-gel sulfonated silica (10 MPa and 49%, respectively) suggests that the SiO*_x_*SO_3_H network lacks long-range order. This is a clear indication of the benefits of performing the sol-gel reaction in fibers during electrospinning, as opposed to allowing for a sol-gel reaction in the blended polymer mixture of a solution-cast film.

### 3.3. Comparison of Membrane Type-A with an Ultra-Low EW PFIA Film and with PFSA/Sulfonated Silica Composite Membranes

A final comparison was made between the proton conductivity of Membrane Type-A and of a homogeneous solution cast membrane composed of neat, low equivalent weight perfluoro imide acid ionomer (660 EW PFIA from 3M Company). This novel ionomer has two ion-exchange sites per sidechain, one sulfonamide and one sulfonic acid. It was developed as an alternative to low EW perfluorosulfonic acid polymers, and has improved crystallinity due to longer CF_2_ runs between side chains along the polymer backbone [10].

**Figure 8:**
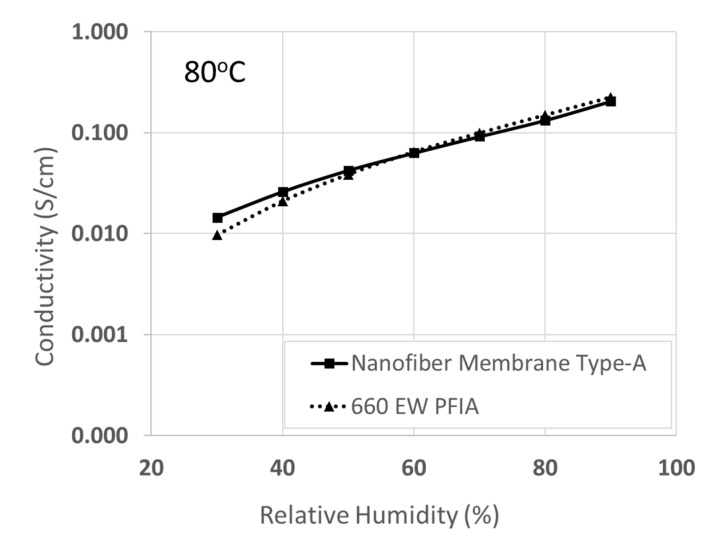
Comparison of in-plane proton conductivity at 80 °C versus relative humidity for hybrid nanofiber Membrane Type-A (65-15/20 PFSA-SiO*_x_*SO_3_H/PVDF, with sol-gel sulfonic acid silica sites in 825 EW PFSA fibers) and a 660 EW PFIA solution-cast membrane.

As can be seen in Figure 8, membrane Type-A exhibits a comparable conductivity to PFIA at 80 °C and high humidity (e.g., 0.21 S/cm vs. 0.22 S/cm, for Type-A and PFIA, respectively, a 90% RH) but has somewhat higher conductivity below 50% RH, e.g., 0.026 S/cm at 40% RH vs. 0.021 S/cm for PFIA. Additional comparative data, including IEC, room temperature swelling and conductivity in water, and tensile strength, for the both membranes are presented in Table 6. Although the two membranes have the same effective ion-exchange capacity, the gravimetric water uptake of the nanofiber film is lower by a factor of 2.2, the in-plane swelling is lower by a factor of 3, and the dry film stress at break is nearly two times greater. All these advantageous characteristics are highly desirable in H_2_/air fuel cell applications. In summary, the combination of data from Table 5 and Figure 8, demonstrate that an electrospun nanofiber composite membrane with a sulfonated silica network added to a moderately low EW PFSA phase is a viable alternative to ultra-low EW perfluorinated ionomer materials for applications in PEM fuel cells.

A comparison of the in-plane proton conductivity of Membrane Type-A and various ionomer membranes referenced in the literature, which contain a cation-exchange ionomer and either sol-gel sulfonated silica or sulfonated polyhedral oligomeric silsesquioxane (sPOSS) is given in Table 7. Data are presented for the conductivity at 80 °C and 50% relative humidity. Two membranes were homogeneous solution-cast films and three were created via nanofiber electrospinning. The proton conductivity of Membrane Type-A compares well with that of all other films. The second highest-conductivity membrane, containing 35 wt.% sPOSS, was not practically stable and degraded over time when immersed in liquid water as sPOSS leached from the sample.

## 4. Conclusions

Hybrid nanofiber composite membranes containing 825 EW PFSA ionomer, reinforcing PVDF fibers, and an inorganic sulfonated silica network were fabricated via dual fiber electrospinning and sol-gel hydrolysis/condensation. The membranes were developed for possible use in H_2_/air fuel cells, but they may also have applications for electrodialysis separation under harsh conditions (e.g., at elevated temperatures). The goal of this work was to increase the proton conductivity of the PFSA membrane especially at low relative humidity and to reduce its lateral swelling in water. Fiber mats were prepared without the sulfonated silica and with the sulfonated silica network either in the PFSA matrix or in the PVDF fibers. The electrospun mats were densified by hot-pressing to soften and fuse the PFSA which filled the void volume between reinforcing PVDF fibers. This led to composite membranes with PVDF fibers embedded in PFSA matrix. A solution cast blend membrane was also prepared and evaluated (as a reference material), where the casting solution contained PFSA, PVDF, and the sol-gel precursor species. The best membrane, in terms of proton conductivity, was made by incorporating the sulfonated silica network in the PFSA matrix (Membrane Type-A) while the lowest conductivity was observed when the sulfonated silica was incorporated into the reinforcing PVDF fibers (Type-B). A Type-A membrane containing 65 wt.% PFSA with embedded sulfonated silica network (15 wt.% overall) and 20 wt.% PVDF reinforcing fibers was superior to a pristine/neat 825 EW PFSA membrane in terms of both the proton conductivity in the 30–90% RH at 80 °C (a 35% increase) and lateral swelling in water (a 68% reduction). In contrast, the properties of a solution cast PFSA/PVDF membrane with sol-gel silica (similar overall composition to that of Membrane Type-A) were quite poor, demonstrating the efficacy of adding sol-gel silica to only the PFSA component of the blend via dual fiber electrospinning. Additionally, it was shown that nanofiber composite Membrane Type-A was superior to a homogeneous film of 660 EW PFIA (perfluoroimide acid ionomer) in terms of lower welling in water, better mechanical strength and slightly better proton conductivity at low relative humidity. Overall, the results demonstrate that the intelligent combination of sol-gel silica doping of a moderately low PFSA ionomer, coupled with nanofiber composite membrane design can produce thin films with excellent properties for fuel cell applications that are superior to ultra-low equivalent weight ionomers. Further testing of these membranes is required, to assess their performance and durability in fuel cell membrane-electrode-assemblies.

## Figures and Tables

**Table 1:** Electrospinning conditions for an 80/20 825 EW PFSA/PVDF, 65-15/20 825EW PFSA-SiO_x_SO_3_H/PVDF, and 65/15-20 825EW PFSA/SiOxSO3H-PVDF composite membranes.

Characteristics of Electrospinning Process	Membrane A		Membrane B		Membrane C	
	Fiber 1	Fiber 2	Fiber 1	Fiber 2	Fiber 1	Fiber 2
	PFSA/SiO*_x_*SO_3_H/PEO	PVDF	PFSA/PEO	PVDF/SiO*_x_*SO_3_H	PFSA/PEO	PVDF
Solution Composition (wt./wt.)	69/30/1	-	99/1	60/40	99/1	-
Voltage (kV)	9.0	9.0	8.0	13.0	8.0	9.0
Solution Flow Rate (mL/h)	0.5	0.16	0.25	0.25	0.5	0.16
Spinneret to Collector Distance (cm)	8.0	8.0	8.0	8.0	8.0	8.0
Relative Humidity (%)	30		30		30	
Membrane Final Composition	65-15/20 PFSA-SiO*_x_*SO_3_H/PVDF		65/15-20 PFSA/SiO*_x_*SO_3_H-PVDF		80/20 PFSA/PVDF	

**Table 2:** Compositions of electrospun raw nanofiber mats and final membranes (after completion of the sol-gel condensation reaction).

Ionomer Fiber Composition Before Sol-Gel Reaction		Ionomer/PVDF Fiber Wt. Ratio	Final Membrane Composition		
825 EW PFSA wt.%	(TEOS + CSPTC) wt.%		825 EW PFSA wt.%	SiO*_x_*SO_3_H wt.%	PVDF wt.%
100	0	80/20	80	0	20
80	20	80/20	71	9	20
70	30	80/20	65	15	20
50	50	80/20	54	26	20

**Table 3:** Hybrid Type-A nanofiber composite membrane properties with varying levels of inorganic sulfonated silica network in PFSA, where all of the membranes contained 20 wt.% PVDF fibers.

Type-A Membranes (PFSA-SiO*_x_*SO_3_H/PVDF)	IEC (mmol/g)	
	Measured	Theoretical
71-9/20	1.37	1.26
65-15/20	1.46	1.45
54-26/20	1.10	1.80
80/20 (Membrane Type-C)	0.96	0.96

**Table 4:** Ionic exchange capacity (IEC), proton conductivity in water at 20 °C, in-plane and gravimetric liquid water swelling for 80/20 PFSA/PVDF, 65-15/20 PFSA-SiO_x_SO_3_H/PVDF, 65/15-20 PFSA/SiO*_x_*SO_3_H-PVDF electrospun composites membranes, a 65/15/20 PFSA/SiO*_x_*SO_3_H/PVDF solution cast blend membrane, and a solution cast neat PFSA membrane. All membrane used 825 EW PFSA.

Membrane Type	Membrane Composition (wt./wt.)	IEC (mmol/g)	Conductivity in Liquid Water at 20 °C (S/cm)	Swelling in Water at 20 °C	
				Gravimetric (%)	In-Plane (%)
Type-A	65-15/20 PFSA-SiO_x_SO_3_H/PVDF	1.46	0.089	49	12
Type-B	65/15-20 PFSA/SiO_x_SO_3_H-PVDF	1.42	0.059	68	12
Type-C	80/20 PFSA/PVDF	0.96	0.087	52	12
Type-D	65-15-20 PFSA-SiO_x_SO_3_H-PVDF	1.44	0.059	23	16
Type-E	Cast 825 EW PFSA	1.21	0.102	47	38

**Table 5:** A summary of tensile characteristics of the four composite membranes in air at 22 °C and ≈20% RH.

Membrane Type	Membrane Composition (wt./wt.)	Stress at Break (MPa)	Strain at Break (%)	Tensile Modulus (MPa)
Membrane Type-A	65-15/20 PFSA-SiO_x_SO_3_H/PVDF	14	134	152
Membrane Type-B	65/15-20 PFSA/SiO_x_SO_3_H-PVDF	18	78	116
Membrane Type-C	80/20 PFSA/PVDF	12	85	138
Membrane Type-D	65-15-20 PFSA-SiO_x_SO_3_H-PVDF	10	49	187

**Table 6:** Comparison of key properties of a solution cast 660 EW PFIA film and a nanofiber composite Type-A membrane (with sulfonated silica in the PFSA matrix).

Membrane Type	IEC (mmol/g)	Conductivity in Water at 25 °C (S/cm)	Gravimetric Swelling in Water at 25 °C (%)	In-Plane Swelling in Water at 25 °C (%)	Stress at Break at 22 °C and 20% RH (MPa)
660 EW PFIA	1.51	0.13	120	35	7
Nanofiber Membrane Type-A	1.46	0.09	49	12	14

**Table 7:** The proton conductivity of ionomer membranes containing sol-gel sulfonated silica or sulfonated polyhedral oligomeric silsesquioxane (sPOSS). In-plane conductivities were measured at 80 °C and 50% relative humidity.

Membrane Type	In-Plane Proton Conductivity (S/cm)	Comments
Membrane Type-A (this work)	0.046	825 EW PFSA with a PFSA/SiO_x_SO_3_H ratio of 65/15 with PVDF reinforcing fibers
Sulfonated poly(arylene ether sulfone) (sPAES) fibers with sPOSS	0.035	2.1 mmol/g IEC sPAES and a sPAES/sPOSS ratio of 60/40, with 30% Norland Optical Adhesive * as the reinforcing polymer (from reference [28])
PFSA fibers with sPOSS	0.083	825 EW PFSA and a PFSA/sPOSS with 26% Norland Optical Adhesive * as the reinforcing polymer (from reference [27])
Solution-cast Nafion with sPOSS	0.040	Nafion with 2% sPOSS, From reference [39]
Solution-cast 1100 EW Nafion with sol-gel sulfonated silica	0.020	1.28 mmol/g membrane IEC, from reference [40]

* Norland Optical Adhesive is a UV-curable polyurethane liquid prepolymer.

## References

[1] Wycisk R., Pintauro P.N., Park J.W. (2014). New developments in proton conducting membranes for fuel cells. Curr. Opin. Chem. Eng..

[2] Kreuer K.-D., Paddison S.J., Spohr E., Schuster M. (2004). Transport in proton conductors for fuel-cell applications: Simulations, elementary reactions, and phenomenology. Chem. Rev..

[3] Alberti G., Casciola M. (2003). Composite Membranes for Medium-Temperature PEM Fuel Cells. Annu. Rev. Mater. Res..

[4] Devanathan R. (2008). Recent developments in proton exchange membranes for fuel cells. Energy Environ. Sci..

[5] Mauritz K.A., Moore R.B. (2004). State of Understanding of Nafion. Chem. Rev..

[6] Fujimura M., Hashimoto T., Kawai H. (1981). Small-angle x-ray scattering study of perfluorinated ionomer membranes. 1. Origin of two scattering maxima. Macromolecules.

[7] Gierke T.D., Munn G.E., Wilson F.C. (1981). The Morphology in Nafion Perfluorinated Membrane Products, as Determined by Wide- and Small- Angle X-ray Studies. J. Polym. Sci. Polym. Phys. Ed..

[8] Maalouf M., Pyle B., Sun C.-N., Wu D., Paddison S.J., Schaberg M., Emery M., Lochhaas K.H., Hamrock S.J., Ghassemi H. (2009). Proton exchange membranes for high temperature fuel cells: Equivalent weight and end group effects on conductivity. ECS Trans..

[9] Yandrasits M.A., Hamrock S.J. (2010). Membranes for PEM Fuel Cells. Fuel Cell Chemistry and Operation.

[10] Yandrasits M.A., Lindell M.J., Hamrock S.J. (2019). New directions in perfluoroalkyl sulfonic acid–based proton-exchange membranes. Curr. Opin. Electrochem..

[11] Dos Santos L., Rose S., Sel O., Maréchal M., Perrot H., Laberty-Robert C. (2016). Electrospinning a versatile tool for designing hybrid proton conductive membrane. J. Membr. Sci..

[12] Sahu A.K., Selvarani G., Pitchumani S., Sridhar P., Shukla A.K. (2007). A Sol-Gel Modified Alternative Nafion-Silica Composite Membrane for Polymer Electrolyte Fuel Cells. J. Electrochem. Soc..

[13] Adjemian K.T., Lee S.J., Srinivasan S., Benziger J., Bocarsly B. (2002). Silicon Oxide Nafion Composite Membranes for Proton-Exchange Membrane Fuel Cell Operation at 80–140 °C. J. Electrochem. Soc..

[14] Pereira F., Vallé K., Belleville P., Morin A., Lambert S., Sanchez C. (2008). Advanced Mesostructured Hybrid Silica−Nafion Membranes for High-Performance PEM Fuel Cell. Chem. Mater..

[15] Yen C.Y., Lee C.H., Lin Y.F., Lin H.L., Hsiao Y.H., Liao S.H., Chuang C.Y., Ma C.C.M. (2007). Sol-gel derived sulfonated-silica/Nafion (R) composite membrane for direct methanol fuel cell. J. Power Sources.

[16] Jiang R., Kunz H.R., Fenton J.M. (2006). Composite silica/Nafion^®^ membranes prepared by tetraethylorthosilicate sol–gel reaction and solution casting for direct methanol fuel cells. J. Membr. Sci..

[17] Kannan A.G., Choudhury N.R., Dutta N.K. (2009). In situ modification of Nafion^®^ membranes with phospho-silicate for improved water retention and proton conduction. J. Membr. Sci..

[18] Sahu A.K., Pitchumani S., Sridhar P., Shukla A.K. (2009). Co-assembly of a nafion-mesoporous zirconium phosphate composite membrane for PEM fuel cells. Fuel Cells.

[19] Hill M.L., Kim Y.S., Einsla B.R., McGrath J.E. (2006). Zirconium hydrogen phosphate/disulfonated poly(arylene ether sulfone) copolymer composite membranes for proton exchange membrane fuel cells. J. Membr. Sci..

[20] Jiang R., Russell Kunz H., Fenton J.M. (2006). Influence of temperature and relative humidity on performance and CO tolerance of PEM fuel cells with Nafion^®^–Teflon^®^–Zr(HPO_4_)_2_ higher temperature composite membranes. Electrochim. Acta.

[21] Silva V.S., Ruffmann B., Silva H., Silva V.B., Mendes a., Madeira L.M., Nunes S. (2006). Zirconium oxide hybrid membranes for direct methanol fuel cells—Evaluation of transport properties. J. Membr. Sci..

[22] Woo M.H., Kwon O., Choi S.H., Hong M.Z., Ha H.-W., Kim K. (2006). Zirconium phosphate sulfonated poly (fluorinated arylene ether)s composite membranes for PEMFCs at 100–140 °C. Electrochim. Acta.

[23] Pica M., Donnadio A., Casciola M., Cojocaru P., Merlo L. (2012). Short side chain perfluorosulfonic acid membranes and their composites with nanosized zirconium phosphate: Hydration, mechanical properties and proton conductivity. J. Mater. Chem..

[24] Kim Y.S., Wang F., Hickner M., Zawodzinski T.A., McGrath J.E. (2003). Fabrication and characterization of heteropolyacid (H_3_PW_12_O_40_)/directly polymerized sulfonated poly(arylene ether sulfone) copolymer composite membranes for higher temperature fuel cell applications. J. Membr. Sci..

[25] Ramani V., Kunz H.R., Fenton J.M. (2006). Metal dioxide supported heteropolyacid/Nafion^®^ composite membranes for elevated temperature/low relative humidity PEFC operation. J. Membr. Sci..

[26] Li L., Wang Y. (2006). Proton conducting composite membranes from sulfonated polyethersulfone Cardo and phosphotungstic acid for fuel cell application. J. Power Sources.

[27] Choi J., Wycisk R., Zhang W., Pintauro P.N., Lee K.M., Mather P.T. (2010). High conductivity perfluorosulfonic acid nanofiber composite fuel-cell membranes. ChemSusChem.

[28] Choi J., Lee K.M., Wycisk R., Pintauro P.N., Mather P.T. (2010). Sulfonated Polysulfone/POSS Nanofiber Composite Membranes for PEM Fuel Cells. J. Electrochem. Soc..

[29] Maneeratana V., Bass J.D., Azaïs T., Patissier A., Vallé K., Maréchal M., Gebel G., Laberty-Robert C., Sanchez C. (2013). Fractal Inorganic−Organic Interfaces in Hybrid Membranes for Efficient Proton Transport. Adv. Funct. Mater..

[30] Laberty-Robert C., Vallé K., Pereira F., Sanchez C. (2011). Design and properties of functional hybrid organic-inorganic membranes for fuel cells. Chem. Soc. Rev..

[31] Dos Santos L., Maréchal M., Guillermo A., Lyonnard S., Moldovan S., Ersen O., Sel O., Perrot H., Laberty-Robert C. (2016). Proton Transport in Electrospun Hybrid Organic-Inorganic Membranes: An Illuminating Paradox. Adv. Funct. Mater..

[32] Ballengee J.B., Pintauro P.N. (2011). Composite fuel cell membranes from dual-nanofiber electrospun mats. Macromolecules.

[33] Choi J., Lee K.M., Wycisk R., Pintauro P.N., Mather P.T. (2010). Nanofiber composite membranes with low equivalent weight perfluorosulfonic acid polymers. J. Mater. Chem..

[34] Ballengee J.B., Haugen G.M., Hamrock S.J., Pintauro P.N. (2013). Properties and Fuel Cell Performance of a Nanofiber Composite Membrane with 660 Equivalent Weight Perfluorosulfonic Acid. J. Electrochem. Soc..

[35] Laforgue A., Robitaille L., Mokrini A., Ajji A. (2007). Fabrication and Characterization of Ionic Conducting Nanofibers. Macromol. Mater. Eng..

[36] Lee K.M., Choi J., Wycisk R., Pintauro P.N., Mather P. (2009). Nafion Nanofiber Membranes. ECS Trans..

[37] Germer W., Harms C., Tullius V., Leppin J., Dyck A. (2015). Comparison of conductivity measurement systems using the example ofnafion and anion exchange membrane. Solid State Ionics.

[38] Park J.W., Wycisk R., Pintauro P.N. (2015). Nafion/PVDF Nanofiber Composite Membranes for Regenerative Hydrogen/Bromine Fuel Cells. J. Membr. Sci..

[39] Del Río C., Morales E., Escribano P.G. (2014). Nafion/sPOSS hybrid membranes for PEMFC. Single cell performance and electrochemical characterization at different humidity conditiions. Int. J. Hydrogen Energy.

[40] Xu K., Chanthad C., Gadinski M.R., Hickner M.A., Wang Q. (2009). Acid-functionalized polysilsesquioxane-nafion composite membranes with high proton conductivity and enhanced selectivity. ACS Appl. Mater. Interfaces.

